# Machine learning for morbid glomerular hypertrophy

**DOI:** 10.1038/s41598-022-23882-7

**Published:** 2022-11-09

**Authors:** Yusuke Ushio, Hiroshi Kataoka, Kazuhiro Iwadoh, Mamiko Ohara, Tomo Suzuki, Maiko Hirata, Shun Manabe, Keiko Kawachi, Taro Akihisa, Shiho Makabe, Masayo Sato, Naomi Iwasa, Rie Yoshida, Junichi Hoshino, Toshio Mochizuki, Ken Tsuchiya, Kosaku Nitta

**Affiliations:** 1 Department of Nephrology, Tokyo Women’s Medical University, 8-1 Kawada-Cho, Shinjuku-Ku, Tokyo, 162-8666 Japan; 2 Clinical Research Division for Polycystic Kidney Disease, Department of Nephrology, Tokyo Women’s Medical University, Tokyo, 162-8666 Japan; 3 Department of Blood Purification, Tokyo Women’s Medical University, Tokyo, 162-8666 Japan; 4 Department of Nephrology, Kameda Medical Center, Chiba, 296-8602 Japan; 5 Japanese Red Cross Saitama Hospital, Saitama, 330-8553 Japan

**Keywords:** Nephrology, Risk factors

## Abstract

A practical research method integrating data-driven machine learning with conventional model-driven statistics is sought after in medicine. Although glomerular hypertrophy (or a large renal corpuscle) on renal biopsy has pathophysiological implications, it is often misdiagnosed as adaptive/compensatory hypertrophy. Using a generative machine learning method, we aimed to explore the factors associated with a maximal glomerular diameter of ≥ 242.3 μm. Using the frequency-of-usage variable ranking in generative models, we defined the machine learning scores with symbolic regression via genetic programming (SR via GP). We compared important variables selected by SR with those selected by a point-biserial correlation coefficient using multivariable logistic and linear regressions to validate discriminatory ability, goodness-of-fit, and collinearity. Body mass index, complement component C3, serum total protein, arteriolosclerosis, C-reactive protein, and the Oxford E1 score were ranked among the top 10 variables with high machine learning scores using SR via GP, while the estimated glomerular filtration rate was ranked 46 among the 60 variables. In multivariable analyses, the *R*^2^ value was higher (0.61 vs. 0.45), and the corrected Akaike Information Criterion value was lower (402.7 vs. 417.2) with variables selected with SR than those selected with point-biserial *r*. There were two variables with variance inflation factors higher than 5 in those using point-biserial *r* and none in SR. Data-driven machine learning models may be useful in identifying significant and insignificant correlated factors. Our method may be generalized to other medical research due to the procedural simplicity of using top-ranked variables selected by machine learning.

## Introduction

Machine learning (ML) refers to computational techniques applied in many fields. ML has been proposed as a valuable and necessary tool for modern healthcare systems^1–4^. Traditional statistics focus on inferring significant risk factors among explanatory variables via model-driven regression using linear models. In contrast, ML focuses on building an accurate predictive model of a target variable from explanatory variables via data-driven regression using non-linear models^2–4^. Statistics uses models with a linear combination of explanatory variables that clearly show each explanatory variable's contribution to the target variable. Comparatively, ML uses non-linear models with more freedom to fit non-linear relationships between explanatory variables and the target variable^4^. Therefore, ML has the potential to discover explanatory variables correlated non-linearly with the target variable that are not detectable with a linear statistical method.

Clinicians are interested in applying ML techniques to the healthcare field^1,5^. However, the modern data-driven approach of ML seems at odds with the traditional model-driven statistical approach in medical research^3,4^. Therefore, a practical method that integrates ML with conventional statistics is needed. Although most ML models are expressed in an implicit form, some ML models are transparent enough to identify novel related factors. ML methods, such as the symbolic regression via genetic programming (SR via GP) technique, have transparency in the created models, allowing an easy understanding of the prediction mechanisms. Furthermore, ML methods can be applied to wide-ranged data (high-dimensional context) because there are no restrictions regarding the number of input variables due to their dimensionality reduction techniques^2,3,6^. Since biological data are often characterized by many variables on few available samples, clinicians wish to ascertain the dominant variables used in the created model rather than the model itself. Even in small studies, SR via GP allows for the exploration of variable importance in the optimized SR model.

Glomerular hypertrophy (GH) (or a large renal corpuscle) reflects the renal injury state^7^. We first reported that GH, defined by maximal glomerular diameter (MaxGD) of ≥ 242.3 μm, implies poor renal prognosis in IgA nephropathy^8^. We confirmed MaxGD as an effectual pathological factor predicting renal IgA nephropathy prognosis in another cohort^9^ and a follow-up study^10^. However, in clinical settings, GH is often underestimated as compensatory GH. The aim of the present study was to investigate the pathophysiological factors associated with GH and to demonstrate that MaxGD, which is quantified histologically by needle biopsy, is not an indicator of compensatory GH but of renal damage itself. Due to difficulties in enrolling an adequate number of cases in renal biopsy studies, a technique is needed to examine small cohorts. Although ML is excellent for analyzing “big data”, techniques for analyzing small sample sizes have also been developed^3,11^. Herein, we integrated traditional statistical methods with ML. We first generated optimized models fitted for the dataset with SR via GP, then selected predominant variables in the models, and subsequently validated them using traditional statistical methods.

## Materials and methods

### Study design

The primary outcome of this study was GH, defined as a MaxGD of ≥ 242.3 μm. This study was approved by the Ethics Committee of the Kameda Medical Center (No. 17–170) and followed the principles of the 1964 Helsinki Declaration. This retrospective study used the same cohort as our previous study (43 patients with IgA nephropathy)^8^. We used passive informed consent (opt-out) for patients. Data were analyzed anonymously.

### Study cohort

**Figure 1:**
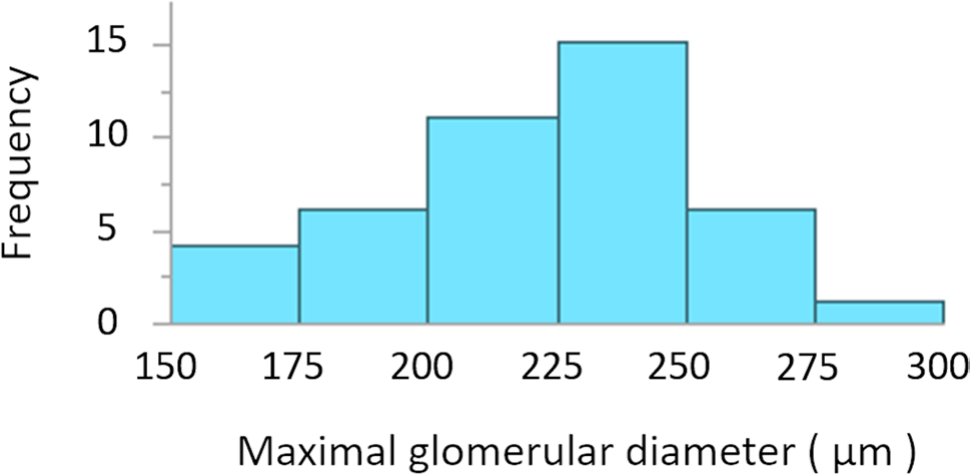
Histogram of MaxGD. The distribution of MaxGD is illustrated as light blue histograms. Abbreviation: MaxGD, maximal glomerular diameter.

We used the same dataset as was used in our previous study^10^. Patient characteristics (median age, 41 years; 26 men and 17 women) were analyzed (Table 1). Figure 1 illustrates a histogram of MaxGD. Fifteen patients (34.9%) had a MaxGD of ≥ 242.3 μm.

**Table 1:** Patient characteristics according to the presence of MaxGD ≥ 242.3 μm: Clinical, laboratory, and pathological findings.

Variables	Entire cohort	Cohort with MaxGD ≥ 242.3 μm	Cohort without MaxGD ≥ 242.3 μm	*P*-value
	n = 43	n = 15	n = 28	
**Clinical findings**				
Height (m)	1.63 (1.47–1.79)	1.64 (1.47–1.79)	1.63 (1.50–1.78)	0.9797
BW (kg)	67.8 (41.6–114.0)	72.9 (54.5–114.0)	62.2 (41.6–90.1)	0.0226
BMI (kg/m^2^)	25.1 (16.8–37.5)	27.8 (21.7–37.5)	23.8 (16.8–32.3)	0.0005
Sex (Men; %)	39.5/60.5	26.7/73.3	46.4/53.6	0.3274
Age (years)	41 (19–59)	41 (26–59)	41 (19–53)	0.6742
Interval from onset (months)	42 (1–325)	37 (1–287)	42 (2–325)	0.6193
SBP (mmHg)	143.1 ± 22.2	145.3 ± 21.2	142.0 ± 23.0	0.6464
DBP (mmHg)	82.3 ± 15.4	84.5 ± 14.8	81.2 ± 15.9	0.3266
MBP (mmHg)	102.6 ± 16.8	104.8 ± 15.9	101.4 ± 17.5	0.4677
PP (mmHg)	60.8 ± 13.4	60.7 ± 14.1	60.8 ± 13.3	0.8285
Edema (%)	86.1/14.0	100.0/0.0	78.6/21.4	0.0764
Acute kidney injury (%)	88.4/11.6	86.7/13.3	89.3/10.7	1.0000
Family history (%)	86.1/14.0	86.7/13.3	85.7/14.3	1.0000
**Laboratory findings**				
WBC (10^3^/µL)	6.78 ± 1.28	7.05 ± 1.23	6.64 ± 1.31	0.3725
Hemoglobin (g/dL)	14.2 ± 2.0	14.6 ± 2.5	14.0 ± 1.7	0.2788
Platelet (10^3^/µL)	21.6 ± 6.1	22.3 ± 7.9	21.2 ± 5.0	0.9898
Total protein (g/dL)	6.48 ± 0.59	6.71 ± 0.51	6.36 ± 0.59	0.1084
Serum albumin (g/dL)	3.79 ± 0.39	3.91 ± 0.40	3.73 ± 0.38	0.1360
eGFR (mL/min/1.73m^2^)	78.6 ± 17.5	72.2 ± 12.9	81.9 ± 18.9	0.1141
C-reactive protein (mg/dL)	0.08 (0.01–0.92)	0.13 (0.01–0.77)	0.06 (0.01–0.92)	0.0313
IgG (mg/dL)	1141.8 ± 321.0	1173.8 ± 401.4	1124.6 ± 275.4	0.9797
IgA (mg/dL)	337.1 ± 139.7	362.1 ± 162.2	323.7 ± 127.2	0.4755
IgM (mg/dL)	166.0 ± 92.0	160.6 ± 84.3	168.9 ± 97.3	0.7832
C3 (mg/dL)	90.4 ± 19.2	103.6 ± 21.6	83.9 ± 14.2	0.0059
C4 (mg/dL)	36.9 ± 10.9	38.7 ± 11.3	36.0 ± 10.8	0.4599
U-Prot (g/day)	1.4 (0.0–7.0)	1.5 (0.6–5.7)	1.1 (0.0–7.0)	0.5493
U-RBC (counts/HPF)	10 (0–200)	1 (0–100)	10 (0–200)	0.0926
U-WBC (counts/HPF)	1.8 (0–100)	1 (0–20)	1 (0–100)	0.9898
Salt intake (g/day)	8.57 ± 3.06	9.04 ± 3.76	8.31 ± 2.64	0.2961
Protein intake (g/kg・IBW/day)	1.05 ± 0.26	1.13 ± 0.31	1.01 ± 0.22	0.0977
**Follow-up findings**				
U-Prot during 10-year follow-up (grades 0–4)	1.1 (0.2–3.4)	1.7 (0.3–3.4)	1.0 (0.2–2.7)	0.0059
Dipstick hematuria during 10-year follow-up (grades 0–4)	1.4 (0.0–3.0)	1.4 (0.1–3.0)	1.4 (0.0–3.0)	0.9289
Increase in U-Prot during 10-year follow-up (%)	62.8/37.2	40.0/60.0	75.0/25.0	0.0236
Increase in dipstick hematuria during 10-year follow-up (%)	60.5/39.5	33.3/66.7	75.0/25.0	0.0077
**Comorbidities**				
Hypertension (%)	23.3/76.7	20.0/80.0	25.0/75.0	1.0000
Hyperuricemia (%)	72.1/27.9	53.3/46.7	82.1/17.9	0.0447
Hypertriglyceridemia (%)	62.8/37.2	46.7/53.3	71.4/28.6	0.1094
Hypercholesterolemia (%)	76.7/23.3	80.0/20.0	75.0/ 25.0	1.0000
Nephrotic Syndrome (%)	86.1/14.0	100.0/0.0	78.6/ 21.4	0.0764
**Histological findings**				
Number of glomeruli	13.4 ± 5.5	12.1 ± 4.5	14.1 ± 5.9	0.1810
Global sclerosis (%)	14.3 (0.0–57.1)	12.5 (0.0–55.6)	14.5 (0.0–57.1)	0.9695
Segmental sclerosis (%)	14.7 (0.0–69.2)	11.1 (0.0–55.6)	17.7 (0.0–69.2)	0.2515
Perihilar hyalinosis (%) [42]	57.1/ 42.9	33.3/66.7	70.4/29.6	0.0269
Crescent (%)	6.3 (0.0–83.3)	6.3 (0.0–33.3)	5.9 (0.0–83.3)	0.6465
Cellular crescent (%)	5.9 (0.0–83.3)	0.0 (0.0–22.2)	5.9 (0.0–83.3)	0.4148
Fibrous crescent (%)	0.0 (0.0–12.5)	0.0 (0.0–12.5)	0.0 (0.0–8.3)	0.3101
Focal adhesions to Bowman's capsule (%) [42]	90.5/ 9.5	80.0/ 20.0	96.3/3.7	0.1220
Thickness of GBM (%) [42]	90.5/ 9.5	86.7/ 13.3	92.6/7.4	0.6080
Mesangial cell proliferation (grades 1–3) 1/ 2/ 3 (%)	20.9/ 32.6/ 46.5	13.3/ 26.7/ 60.0	25.0/ 35.7/ 39.3	0.5353
Number of mesangial cells per mesangial lesion	7 (4–10)	8 (4–10)	6.5 (4–9)	0.3061
Interstitial fibrosis (%)	5.0 (0.0–60.0)	5.0 (0.0–60.0)	5.0 (0.0–30.0)	0.7766
Interstitial inflammation (%)	5.0 (0.0–30.0)	5.0 (0.0–25.0)	5.0 (0.0–30.0)	0.5248
Intimal thickening of interlobular artery (grades 0–3) 0/1/2/3 (%)	48.8/34.9/16.3/ 0.0	20.0/53.3/26.7/0.0	64.3/25.0/10.7/0.0	0.0201
Arteriolosclerosis (grades 0–3) 0/1/2/3 (%)	25.6/53.5/16.3/4.7	6.7/46.7/33.3/13.3	35.7/57.1/7.1/0.0	0.0073
Japanese histological grades (grades 1–4) 1/2/3/4 (%)	4.7/4.7/86.1/4.7	6.7/6.7/80.0/6.7	3.6/3.6/89.3/3.6	1.0000
Oxford M1 (%)	18.6/81.4	20.0/80.0	17.9/82.1	1.0000
Oxford E1 (%)	46.5/ 53.5	40.0/60.0	50.0/50.0	0.5309
Oxford S1 (%)	18.6/ 81.4	26.7/73.3	14.3/85.7	0.4188
Oxford T 0/ 1/ 2 (%)	95.4/2.3/2.3	93.3/0.0/6.7	96.4/3.6/0.0	0.5814
Oxford C 0/ 1/ 2 (%)	44.2/46.5/9.3	46.7/53.3/0.0	42.9/42.9/14.3	0.4128
Localization of glomerulus examined for MaxGD in renal cortex; inner/middle/outer (%)	23.3/55.8/20.9	20.0/60.0/20.0	25.0/53.6/21.4	1.0000
MaxGD (µm)	221.7 ± 30.8	253.1 ± 10.9	204.9 ± 24.0	< 0.0001

Continuous variables are expressed as means ± standard deviation or median (minimum–maximum). Count data are expressed as %. For the variables with missing data, the number of patients with non-missing data are shown in []. Abbreviations: MaxGD, maximal glomerular diameter; n, number; BW, body weight, BMI, body mass index; %, percentages; SBP, systolic blood pressure; DBP, diastolic blood pressure; MBP, mean blood pressure; PP, pulse pressure; WBC, white blood cells; eGFR, estimated glomerular filtration rate; IgG, immunoglobulin G; IgA, immunoglobulin A; IgM, immunoglobulin M; C3, complement component 3; C4, complement component 4; U-Prot, Urinary protein excretion; U-RBC, urinary red blood cells; HPF, high-power field; IBW, ideal body weight; GBM, glomerular basement membrane; M, mesangial hypercellularity; E, endocapillary hypercellularity; S, segmental glomerulosclerosis; T, tubular atrophy/interstitial fibrosis; C, cellular/fibrocellular crescents.

### Variables examined for GH presence

The 60 surveyed predictive variables for GH (56 baseline and four follow-up variables) included patient baseline data, laboratory test data, comorbidities, pathological findings, and medical history. The Supplemental Methods describe the comorbidity definitions and histological kidney biopsy assessment. Table 1 shows the survey items.

### Statistical analyses

Continuous variables are reported as means and standard deviations or as medians (minimum–maximum). Categorical variables are reported as percentages unless stated otherwise. Group differences were evaluated using the unpaired t-test, Mann–Whitney U test, chi-square test, or Fisher’s exact test, as appropriate. Based on a previous report^8^, we classified patients into two groups: those with a MaxGD ≥ 242.3 μm and those with a MaxGD < 242.3 μm. The correlations between MaxGD ≥ 242.3 μm and the other variables were assessed using the point-biserial correlation coefficient (point-biserial *r*). Multivariable linear regression analyses were also performed for MaxGD (μm).

### ML with SR via GP

To create an ML-based predictive model (based on MaxGD ≥ 242.3 μm presence), a dichotomous response variable (1 or 0) and 60 explanatory variables were used. We first performed a permutation test^12,13^ as a preprocessing test for the dataset to control Type-I errors and to verify meaningful relationships between explanatory variables and the target variable^14^. We used the Python scikit-learn library^15^ (Supplemental Methods) for the entire dataset. We used DataModeler version 9.3 (Evolved Analytics LLC, Rancho Santa Fe, CA, USA) that runs on Mathematica version 12.1 (Wolfram Research Incorporated, Champaign, IL, USA) to create a dichotomous discriminant function for predicting MaxGD ≥ 242.3 μm. DataModeler performs SR via GP, a type of ML, which was evaluated by the leave-one-out cross-validation (LOO-CV) procedure in accordance with the study sample size^11–13^. SR via GP is a regression method based on evolutionary algorithm searching in the function space for explicit mathematical formulas through accuracy/complexity trade-off^16^. SR is flexible enough to fit almost any relationships between explanatory variables and the target variable by simultaneously searching both the forms of functions and the values of parameters. Initial mathematical expressions are formed by a random combination of mathematical building blocks such as variables, coefficients, constants, and mathematical operators, including + , − , × , ÷ , and *√*. Then, they are randomly evolved by adding new building blocks, deleting some blocks, or mutating some parts until the desired accuracy and simplicity of the models are attained. In the process of generating thousands of these models, pathological ones are extinguished, while eugenic ones are further evolved. Finally, models lining the Pareto front in the function space are selected, and the optimized model is created as their trimmed mean. Since the obtained formulas (both accurate and simple) are described in conventional arithmetic expressions using variables in the dataset, their behaviors can be explored by numerical simulations^17^.

### Procedures for producing prognostic, predictive functions, and ML scores for GH presence

We then define the ML score (GP) of each explanatory variable from a ratio of the number of models that use it to the total number of appropriate models generated by SR via GP. Each ratio is multiplied by 100 so that all the scores sum up to 100. The ML score is, therefore, a number between 0 and 100 assigned to each explanatory variable that represents its contribution in predicting the target variable and belongs to a kind of feature importance^18^ in ML.

In detail, by using SR via GP, we generated four independent evolution species per cross-validation and accumulated 43 series of predictive models in LOO-CV. The best result with the highest AUC of ROC curve was chosen from 10 consecutive LOO-CV trials. LOO-CV is a method in which only one case is extracted from the sample data as a test case, and the rest are used as training data. By repeating this for all cases, all cases were tested. LOO-CV is a type of *K*-fold cross-validation, with *K* equal to the sample size, that is suitable for smaller sample sizes^11^. During this process, a reduction in the dimensionality of 60 predictive variables was performed by a capability intrinsic to SR via GP. We selected approximately the top 10% of the suitable models that were neither overfitting nor underfitting through the trade-off between lower model complexity and lower 1–*R*^*2*^ or error (*R*^*2*^: coefficient of determination). The complexity indicates overfitting, and 1 − *R*^*2*^ indicates the degree of underfitting. Then, we examined the appearance frequency of predictive variables in the suitable models and determined the dominant predictive variables that were utilized in more than 5% of the selected top 10% models. At this stage, we counted the appearance frequencies [Frequencies (GP), range 0 to 100] of each variable, representing variable importance in relation to the study outcome using SR via GP. Similarly, the ML score by SR via GP [ML scores (GP), range from 0 to 100] of each variable was defined as the number of models that used the variable divided by the total number of suitable models and multiplied by 100. The ML scores by other types of machine learning techniques or statistical methods^18^ were calculated similarly so that the sum of ML scores of all the variables was 100.

### Comparison of classification capabilities and variable importance between SR via GP and other ML models

We also conducted statistical comparisons of classification capabilities between SR via GP and typical ML models. Other ML models included simple linear regression, Lasso regression, Ridge regression, naïve Bayes, logistic regression, Support Vector Machine, Random Forest, and eXtreme Gradient Boosting (XGBoost). Using the same dataset and LOO-CV, statistical metrics for classification capabilities, including area-under-the-receiver-operating-characteristic-curve (AUC-ROC), accuracy, precision, recall, and F1 score, were compared. Any ML model with hyperparameters was optimized according to its conventional procedure, using Optuna (Preferred Networks Inc., Tokyo, Japan), an automatic hyperparameter optimization software for machine learning (https://optuna.org/).

Then, we compared our ML score with other types of variable importance in the classification of MaxGD ≥ 242.3 μm, including six types of statistical or machine learning models. They were F-statistic (ANOVA), Maximal Information Coefficient, Impurity Reduction with Random Forest, Split Count with XGBoost, Coverage with XGBoost (the number of observations affected by the split), and permutation test with Random Forest. The average value of seven kinds of variable importance, including that by SR, was also figured out.

### Statistical assessment

Using the ML scores associated with MaxGD ≥ 242.3 μm derived from SR via GP, we compared variables selected by point-biserial *r* and from ML scores. Multivariable logistic regression analyses and multivariable linear regression analyses related to MaxGD were used to validate discriminatory ability, goodness-of-fit, and collinearity. The model’s goodness-of-fit was assessed using R-squared (*R*^2^), McFadden’s pseudo-R-squared (pseudo-*R*^2^)^19^, and the corrected Akaike Information Criterion (AIC)^20^. The model’s discriminatory ability was evaluated using the concordance statistic (C-statistic)^21,22^, and multicollinearity was assessed using the variance inflation factor (VIF)^23^. We used standard methods to estimate the sample sizes needed for the multivariate logistic and linear regression analyses; at least five positive outcomes were needed for each included independent variable^24–26^. Two-tailed statistical significance was set at *P* < 0.05. Analyses were performed using JMP Pro version 15.1.0 (SAS Institute, Cary, NC, USA), and the remaining analyses were performed using Mathematica version 12.1 (Wolfram Research Inc., Champaign, IL, USA), DataModeler and Python version 3.7 using modules and libraries from Scikit-learn (https://scikit-learn.org).

### Ethical approval

This study was approved by the Ethics Committee of the Kameda Medical Center (No. 17–170) and followed the principles of the 1964 Helsinki Declaration.

### Informed consent

We used passive informed consent (opt-out) for patients, and all data were analyzed anonymously.

## Results

### Permutation test with the dataset

**Figure 2:**
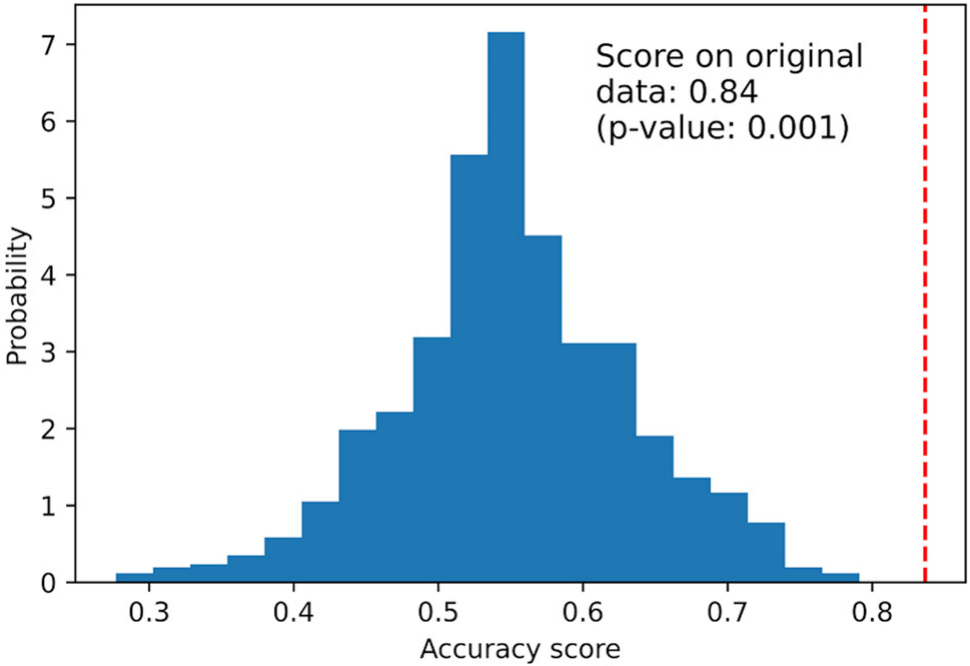
Permutation test results with the original dataset (permutation test scores for the classifier of MaxGD ≥ 242.3 μm). The distribution of accuracy score for the permuted data is illustrated as blue histograms. It represents the result of 5000 permutation tests for assessing classifier performance when selecting the 60 most discriminative variables. The red dotted line indicates the accuracy score value (0.84) obtained by the classifier in the original dataset (permutation P-value, 0.001). Abbreviation: GD, glomerular diameter.

Figure 2 shows the distribution of accuracy scores in the permutation test. The accuracy score with the original dataset (MaxGD dataset) was 0.84, significantly higher than that obtained using permuted datasets, and the P-value of the permutation test was 0.001. This indicated a low likelihood of this score being obtained by chance alone. It provides evidence that the MaxGD dataset contains a dependency between explanatory and target variables, with reliable classification performance and sufficient information to be analyzed.

### Correlation coefficients (point-biserial Rs) between predictive variables and MaxGD ≥ 242.3 μm

All correlation coefficients (denoted as point-biserial *R*) of prognostic, predictive variables in the presence of MaxGD are shown (Supplemental Fig. 1). Further, the point-biserial *R* values of the top 20 variables with the strongest positive or negative correlations are listed (Supplemental Table 1).

### Analysis of predictive variables for MaxGD ≥ 242.3 μm using ML

**Figure 3:**
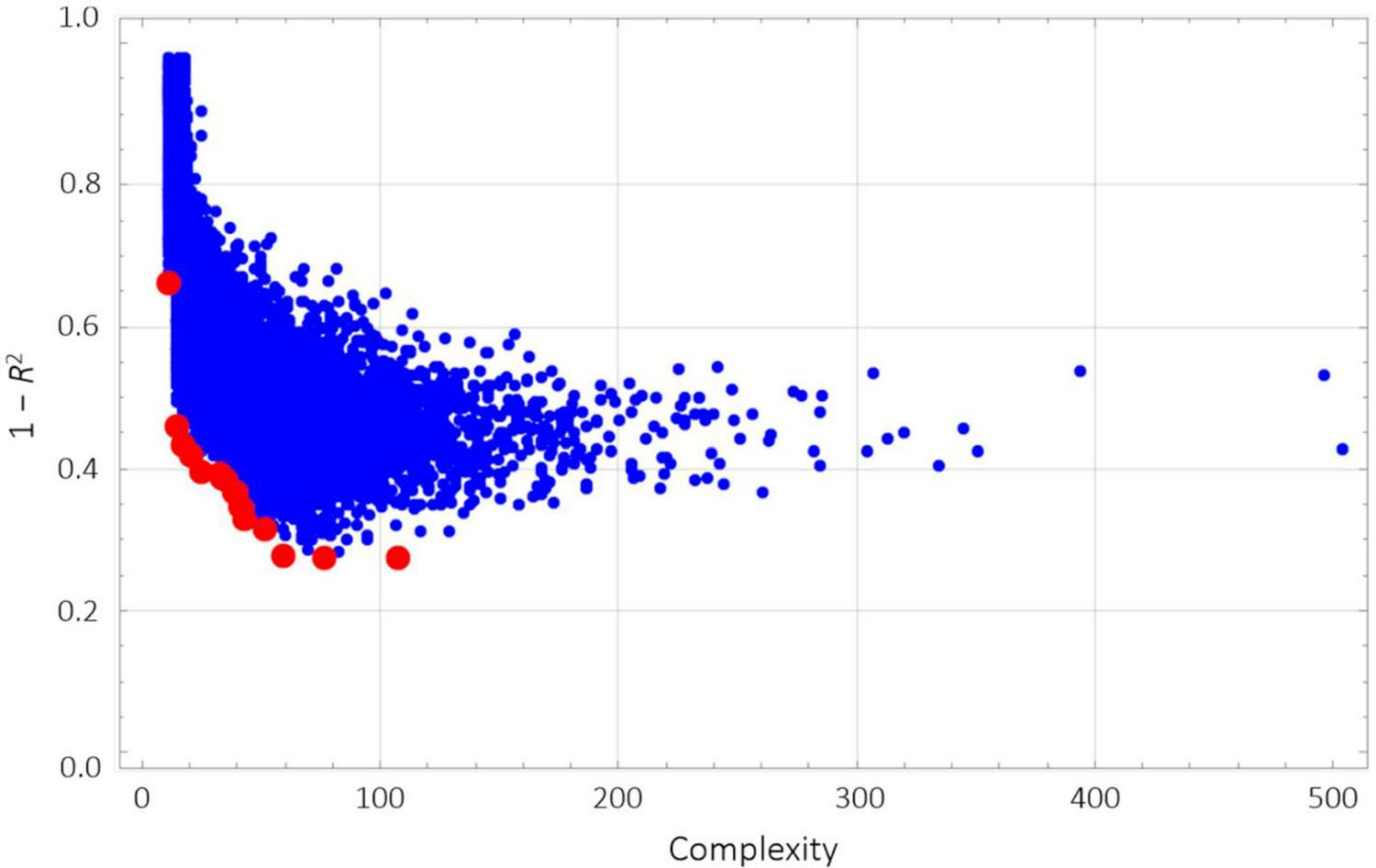
Distribution of functions generated with symbolic regression via genetic programming. Generated functions are plotted on the function space, where the horizontal axis represents the complexity of a function and the vertical axis represents 1 − *R*^2^ or error. In total, 19,437 predictive functions are generated with symbolic regression via genetic programming. Each dot represents one function, and the red dots represent functions on the Pareto front that are candidates for optimized functions with ensemble learning.

**Figure 4:**
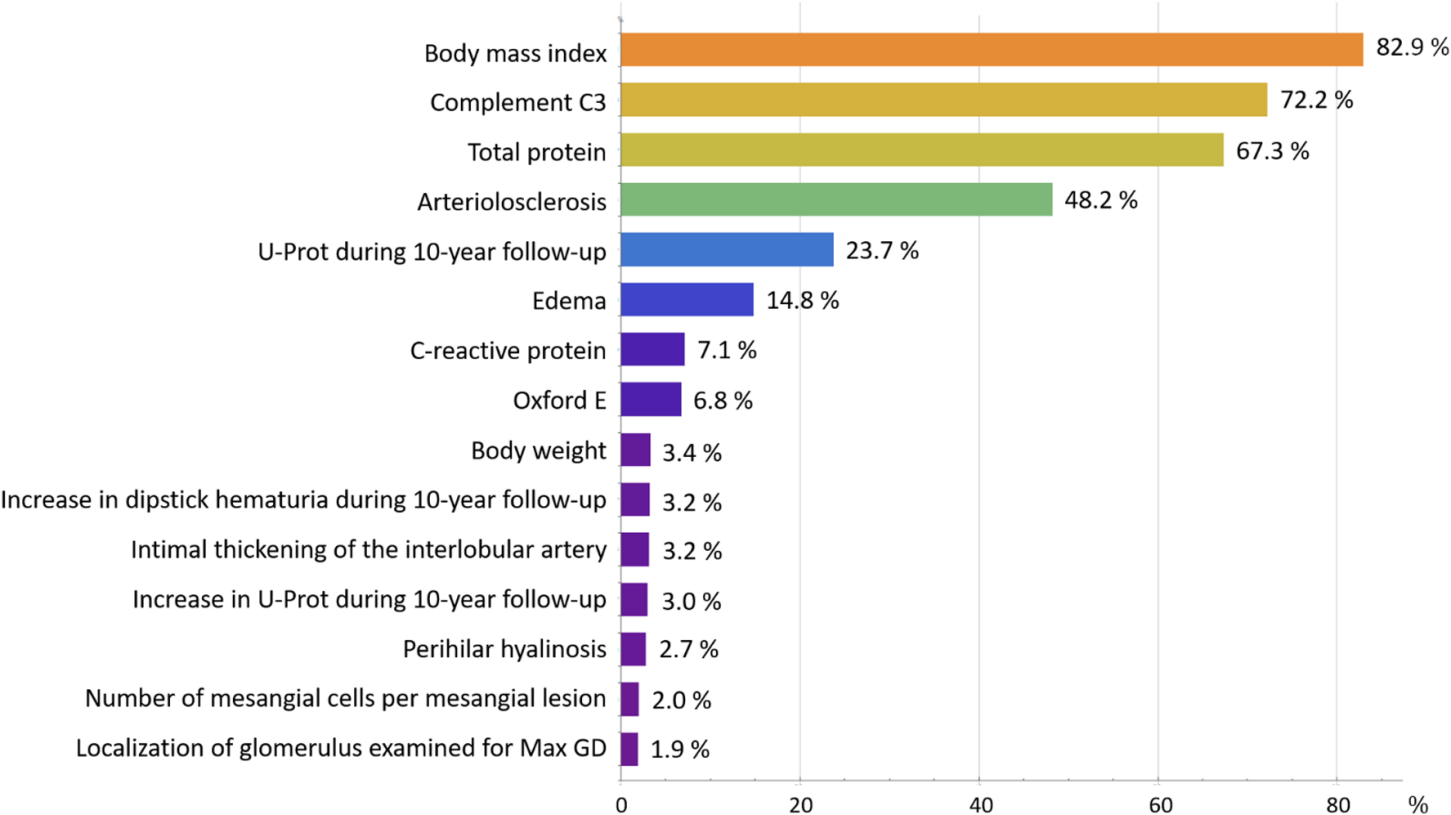
Frequencies (GP): Frequently utilized predictive variables in selected models using SR via GP. The 15 most frequently utilized predictive variables in 1819 predictive functions, which are selected among 19,437 models generated in the leave-one-out cross-validation using symbolic regression via genetic programming, are listed in descending order. The horizontal axis represents the appearance frequencies [Frequencies (GP)]: the percentage at which each predictive variable is utilized in all 1,819 predictive functions. Abbreviations: GP, genetic programming; SR, symbolic regression; C3, component 3; U-Prot, urinary protein excretion; Oxford E1, the presence of endocapillary hypercellularity; MaxGD, maximal glomerular diameter.

We generated 19,437 functions using GP, and Fig. 3 shows their distribution in the function space. We selected 1,819 functions with lower complexities and lower 1 − *R*^2^ in each epoch to limit the number of models to 8%–20% of all generated models. We ranked the **F**requencies **(GP)** of all predictive variables in the 1,819 limited functions (Supplemental Fig. 2). The top 15 variables and their **F**requencies **(GP)** are shown (Fig. 4).

### ML score

Table 2 shows the top 15 variables with high Frequencies (GP) and ML scores (GP) in association with MaxGD ≥ 242.3 μm. The rankings of ML scores (GP) and Frequencies (GP) have the same results. The Frequencies (GP) values, ML score (GP) values, ML score (GP) ranks, *|R|* values, and |*R*| ranks for each variable are listed (Table 2). The top 8 variables with a higher ML score (GP) were body mass index (BMI), complement C3, serum total protein, arteriolosclerosis, urinary protein excretion during the 10-year follow-up, edema, C-reactive protein, and the Oxford E1 score. The estimated glomerular filtration rate (eGFR) (15th in the *|R|* rank) was not in the top 15 rankings in the ML scores (GP) (46th rank), indicating that MaxGD is not affected by nephron disappearance but by another mechanism. Inflammatory indicators, such as C-reactive protein (16th in the *|R|* rank), Oxford E1 (41st in the *|R|* rank), and the number of mesangial cells per mesangial lesion (25th in the *|R|* rank) ranked in the top 15 ML scores (GP), indicating an association between MaxGD and inflammation.

**Table 2:** Top 15 variables in machine learning score via genetic programming [ML score (GP)] in association with the MaxGD ≥ 242.3 μm.

Variables	Frequencies (GP)	ML score (GP)	Rank of ML score (GP)	*|R|*	Rank of |*R*|
BMI (kg/m^2^)	82.9	22.2	1	0.53	1
Complement C3 (mg/dL)	72.2	19.4	2	0.47	3
Total protein (g/dL)	67.3	18.1	3	0.29	13
Arteriolosclerosis (grades 0–3)	48.2	12.9	4	0.50	2
U-Prot during 10-year follow-up (grades 0–4)	23.7	6.4	5	0.46	4
Edema (vs. no)	14.8	4.0	6	0.30	11
C-reactive protein (mg/dL)	7.1	1.9	7	0.26	16
Oxford E1 (vs. no)	6.8	1.8	8	0.10	41
Body weight (kg)	3.4	0.9	9	0.41	5
Increase in dipstick hematuria during 10-year follow-up (vs. no)	3.2	0.9	10	0.41	6
Intimal thickening of the interlobular artery (grades 0–3)	3.2	0.9	11	0.39	7
Increase in U-Prot during 10-year follow-up (vs. no)	3.0	0.8	12	0.35	9
Perihilar hyalinosis (vs. no)	2.7	0.7	13	0.37	8
Number of mesangial cells per mesangial lesion	2.0	0.5	14	0.15	25
Localization of glomerulus examined for MaxGD (inner/middle/outer)	1.9	0.5	15	0.03	52

The Frequencies (GP) values, The ML score (GP) values, ML score (GP) ranks, |*R*| values, and |*R*| ranks for each variable are listed. The rankings in ML scores (GP) and Frequencies (GP) have the same results. *|R|* is the absolute value of point-biserial *r* (Supplemental Fig. 1). Abbreviations: ML, machine learning; GP, genetic programming; MaxGD, maximal glomerular diameter; point-biserial *r*, point-biserial correlation coefficient; BMI, body mass index; C3, component 3; U-Prot, Urinary protein excretion; Oxford E1, the presence of endocapillary hypercellularity.

### Comparison of classification capabilities and variable importance between SR via GP and other ML models

**Figure 5:**
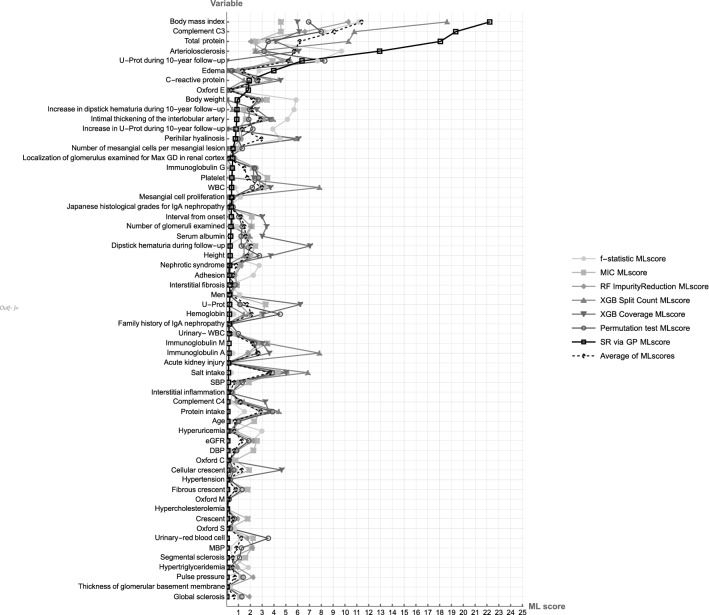
ML scores using eight machine learning models. ML scores of 60 variables. Abbreviations: ML, machine learning; MIC, maximal information coefficient; RF ImpurityReduction, impurity reduction with random forest; XGB, eXtreme Gradient Boosting; SR via GP, symbolic regression via genetic programming; MaxGD, maximal glomerular diameter; eGFR, estimated glomerular filtration rate; WBC, white blood cell; U-Prot, Urinary protein excretion; SBP, systolic blood pressure; MBP, mean blood pressure; DBP, diastolic blood pressure; Complement C4, complement component 4; Complement C3, complement component 3.

Finally, the classification capabilities of nine ML models are shown in Table 5. SR via GP revealed the highest F1-score of 0.615, indicating that it has a well-balanced predictive capability in precision and recall (i.e., sensitivity), with the highest accuracy of 0.767. Regarding AUC-ROC, SR via BP showed the second-best score. Figure 5 shows the seven kinds of ML scores of 60 variables with their average value. The ML score by SR via GP demonstrated well-focused scores compared to others. BMI, C3C, TP, and arteriosclerosisG03 were the most elevated of all the ML scores.

### Validation using a conventional statistical technique

Multivariable logistic regression analyses and multivariable linear regression analyses related to MaxGD were performed to validate discriminatory ability, goodness-of-fit, and collinearity for comparing the variables selected from the ML score (GP) with those selected from point-biserial *r*.

Table 3 shows the results of multivariable logistic regression analyses for MaxGD ≥ 242.3 μm using the top three variables based on the ML score (GP) or |*R*|. Although the model based on the top three variables of the ML score (GP) (Model ML top 3) detected two significant variables, BMI and serum total protein, the model based on the top three variables of the |*R*| (Model |*R*| top 3) detected no significant variables. Furthermore, the values of pseudo-*R*^2^ and C-statistic were higher (0.50 vs. 0.42 and 0.93 vs. 0.87) and the AIC value was lower (36.9 vs. 41.5) in the Model ML top 3 than in the Model |*R*| top 3.

**Table 3:** Multivariable logistic regression analyses for the MaxGD ≥ 242.3 μm using top 3 variables based on ML score (GP) or |*R*|

Model based on top 3 variables of ML score (GP) pseudo-*R*^2^ = 0.50, AICc = 36.9, C-statistic = 0.93	Odds ratio (95% CI)	*P* value
BMI (1 kg/m^2^ increase)	1.57 (1.11–2.20)	0.0100*
Complement C3 (1 mg/dL increase)	1.05 (0.98–1.13)	0.1393
Total protein (1 g/dL increase)	12.94 (1.68–99.71)	0.0140*

Model based on top 3 variables of |*R*| pseudo-*R*^*2*^ = 0.42, AICc = 41.5, C-statistic = 0.87	Odds ratio (95% CI)	*P* value
BMI (1 kg/m^2^ increase)	1.25 (0.92–1.68)	0.1516
Arteriolosclerosis (grades 0–3)	3.51 (0.93–13.21)	0.0631
Complement C3 (1 mg/dL increase)	1.06 (1.00–1.13)	0.0681

*P < 0.05. Variables of top 3 variables based on ML score (GP) or |R| were included in the multivariate model. Abbreviations: MaxGD, maximal glomerular diameter; ML, machine learning; GP, genetic programming; *|R|*, the absolute value of point-biserial correlation coefficient; pseudo-*R*^2^, McFadden’s pseudo-*R*-squared; AICc, small-sample corrected Akaike Information Criterion; BMI, body mass index; C3, component 3.

Table 4 shows the results of the multivariable linear regression analyses for MaxGD ≥ 242.3 μm using the top eight variables based on the ML score (GP) or |*R*|. Although the Model ML top 8 detected four significant variables, i.e., BMI, serum total protein, C-reactive protein, and the Oxford E1 score, the Model |*R*| top 8 detected no significant variables (Table 4). In the Model ML top 8, the *R*^2^ value was higher (0.61 vs. 0.45), and the AIC value was lower (402.7 vs. 417.2) than that in the Model |*R*| top 8. Furthermore, in Model |*R*| top 8, there were two variables with VIF > 5 and four variables with VIF > 1.81 [calculated by 1/(1-overall model *R*^2^)]^27^, indicating multicollinearity in the model. In contrast, in the Model ML top 8, there were no variables with VIF > 5 or VIF > 2.54 [calculated by 1/(1-overall model *R*^2^], indicating reduced multicollinearity in the model.

**Table 4:** Multivariable linear regression analyses for the MaxGD using top 8 variables based on ML score (GP) or |*R*|

Model based on top 8 variables of ML score (GP) *R*^*2*^ = 0.61, AICc = 402.7	*β*	VIF	*t*-ratio	*P* value
BMI (1 kg/m^2^ increase)	0.47	2.04	3.06	0.0043*
Complement C3 (1 mg/dL increase)	0.09	1.42	0.69	0.4956
Total protein (1 g/dL increase)	0.30	1.73	2.13	0.0401*
Arteriolosclerosis (grades 0–3)	− 0.05	1.76	− 0.36	0.7221
U-Prot during 10-year follow-up (grades 0–4)	0.25	1.58	1.84	0.0747
Edema (vs. no)	− 0.24	1.88	− 1.62	0.1150
C-reactive protein (1 mg/dL increase)	0.23	1.10	2.07	0.0464*
Oxford E1 (vs. no)	0.27	1.23	2.23	0.0327*

Model based on top 8 variables of |*R*| *R*^2^ = 0.45, AICc = 417.2	*β*	VIF	*t*-ratio	*P* value
BMI (1 kg/m^2^ increase)	0.60	6.53	1.84	0.0745
Arteriolosclerosis (grades 0–3)	0.11	2.33	0.55	0.5875
Complement C3 (1 mg/dL increase)	0.11	1.47	0.70	0.4887
U-Prot during 10-year follow-up (grades 0–4)	0.22	1.76	1.27	0.2118
Body weight (1 kg increase)	− 0.39	5.74	− 1.28	0.2096
Increase in dipstick hematuria during 10-year (vs. no)	− 0.01	1.63	− 0.04	0.9688
Intimal thickening of the interlobular artery (grades 0–3)	0.14	1.85	0.82	0.4172
Perihilar hyalinosis (vs. no)	0.15	1.39	0.97	0.3413

*P < 0.05. Top 8 variables based on ML score (GP) or *|*point-biserial *r|* were included in the multivariate model. Abbreviations: MaxGD, maximal glomerular diameter; ML, machine learning; GP, genetic programming; *|R|*, the absolute value of point-biserial correlation coefficient; *R*^2^, *R*-squared; AICc, small-sample corrected Akaike Information Criterion; ***β***, standardized partial regression coefficient; VIF, variance inflation factor; BMI, body mass index; C3, component 3; U-Prot, Urinary protein excretion; Oxford E1, the presence of endocapillary hypercellularity.

## Discussion

This exploratory observational study aimed to elucidate risk factors associated with a maximal glomerular diameter of ≥ 242.3 μm using a generative machine learning technique. We applied the advantages of ML to research using traditional statistical methods and obtained two main findings. First, the increased *R*^2^, pseudo-*R*^2^, C-statistic values, and the decreased AIC values observed in variable selection by ML compared with that observed in variable selection by point-biserial *r* suggest that ML techniques can handle large variable numbers and help elucidate novel factors related to multifactorial diseases. Second, the weak association between MaxGD and eGFR demonstrates that MaxGD represents morbid GH rather than compensatory GH.

Traditional statistics based on a priori knowledge with a hypothesized model may delay clinical research progress as few novel prognostic variables are addressed in each study^3^. Contrastingly, ML has the power to reduce the dimensionality of variables^3,4^, which is based on its intrinsic ability to formulate a predictive data-based model. Performing ML first allows us to account for potential linear and non-linear predictors without unconscious bias, avoiding a priori choice among potential predictors. Nonetheless, ML methods neither assess statistical inference nor offer valid inferences on variable importance; therefore, statistical testing should be performed. Considering that the advantages of ML techniques are flexibility and lack of a priori assumptions about models and that the advantages of traditional statistical approaches are simplicity and transparency of understanding^3,4^, the order of our analysis in the present study was first to narrow down the number of variables by ML and subsequently validate the variables by traditional statistics, such as *R*^2^, pseudo-*R*^2^, AIC, and C-statistic. To overcome the overfitting problem^11^ and generalize the ML results, we focused on the ability of ML to select important variables instead of focusing on its predictive models.

Furthermore, while traditional regression analyses suffer from multicollinearity in the presence of many variables, ML techniques alleviate collinearity limitations by leveraging penalization approaches^28,29^. Interestingly, the variables selected by the ML method showed lower VIFs than those selected by point-biserial *r*, indicating that SR via GP is effective in avoiding the introduction of collinear variables. SR via GP^30,31^ is a versatile and heuristic model that allows detailed analyses, even in datasets with a small sample size, partly because it can control the complexity of models to prevent overfitting of the training data. We confirmed that SR via GP is useful for identifying new risk factors and discriminating against unrelated factors. As we routinely perform SR as the ML method, we used this technique. SR showed excellent results, given variable selection among the ML methods examined (Fig. 5, Table 5).

**Table 5:** Comparison of statistical metrics between nine machine learning models.

Machine learning model	AUC	Accuracy	Precision	Recall	F1-score
Linear Regression	0.536	0.488	0.333	0.467	0.389
Lasso Regression	0.736	0.698	0.563	0.600	0.581
Ridge Regression	0.695	0.651	0.500	0.533	0.516
Logistic Regression	0.662	0.721	0.636	0.467	0.539
Naïve Bayes	0.548	0.512	0.385	0.667	0.488
SVM	0.615	0.721	0.800	0.267	0.400
Random Forest	0.631	0.721	0.714	0.333	0.454
XGBoost	0.826	0.698	0.563	0.600	0.581
Symbolic regression via GP	0.774	0.767	0.727	0.533	0.615

Abbreviations: AUC, area under the curve of receiver operating characteristic curve; SVM, support vector machine; XGBoost, eXtreme Gradient Boosting; GP, genetic programming.

Although GH is multifactorial and occurs in different pathophysiological conditions^7^, it is technically difficult to distinguish true valuable risk factors from the many risk factors using a conventional statistical method. However, the significant power of dimensionality reduction via ML makes such discrimination possible by creating rankings for predictive variables of patients with multifactorial chronic diseases^17^. Herein, the ML score (GP) for MaxGD ≥ 242.3 μm identified the top 8 variables, including BMI, complement C3, serum total protein, arteriolosclerosis, urinary protein excretion (U-Prot) during the 10-year follow-up, edema, C-reactive protein, and the Oxford E1 score. Furthermore, eGFR was ranked 46 in the ML scores (GP), indicating that MaxGD is more relevant for current injuries, such as vascular damage, inflammation, and obesity, rather than past injuries represented by nephron disappearance.

Aging is associated with nephron loss/low eGFR; however, it remains controversial whether these findings are pathological or not^32–35^. Therefore, it is desirable to distinguish compensatory hypertrophy due to nephron loss/low GFR from morbid hypertrophy due to disease activity^7^. The key to understanding this question is the rightward shift^36^ in the glomerular size distribution caused by nephron loss/low GFR and the threshold for morbid GH^7,36^. The five-sixths nephrectomized (or subtotal nephrectomized) model is a frequently used animal model of progressive kidney failure by nephron loss/low GFR. The glomerular diameter in subtotal nephrectomized rats increased to approximately 1.5 times the glomerular diameter of the control group. In comparison, that in the non-nephrectomized rats increased to approximately 1.1 times the glomerular diameter of the control group^37^. Therefore, GH > 1.5 times its original diameter (2.25 times area and 3.38 times volume, assuming glomeruli are spherical) may be morbid^7^. In a recent study examining individual glomerular size using magnetic resonance imaging-based glomerular morphology in a mouse model^38^, the increase in glomerular volume due to aging showed a rightward shift in the glomerular size distribution in the range of < 3 times the volume. Similar results have been observed in humans^33^. Furthermore, while American women with low nephron numbers did not demonstrate GH, American men with low nephron numbers showed marked GH^39^. Thus, glomerular size could be a more direct indicator of disease severity compared with nephron loss.

To the best of our knowledge, our study is the first to identify predictive variables for MaxGD using ML. Our research's methodological novelty included combining ML's exploratory strengths with the validation strengths of conventional statistical methods, and our findings will contribute significantly to future clinical research. Furthermore, our findings on the discrimination between compensatory GH caused by nephron loss and pathological GH have significant clinical importance in nephrology.

One limitation of this study is that it was observational in nature; any observed association does not prove causality. Although countermeasures for small sample sizes^12,13^ were adopted, such as permutation tests and LOO-CV using SR via GP, the small sample size should be noted. Furthermore, since the comparison of superiority and inferiority between ML methods was not the primary focus, we did not adopt the approach of comparing the hit rates of predictive models created by ML. Finally, although one of the strengths of ML, such as SR via GP, is that it allows the exploration of non-linear relationships, complete verification may not be possible with conventional statistical methods when non-linear factors are detected by ML. However, the study results, such as the increase of pseudo-*R*^2^ and *R*^2^ in the model based on factors selected by SR, indicate that the factor selection ability of ML is also excellent for linear factors.

In conclusion, ML with SR via GP demonstrated a weak association between MaxGD and eGFR, indicating that MaxGD represents morbid GH rather than compensatory GH. Furthermore, in a comparative validation using a conventional statistical technique, variable selection by ML avoided collinearity and increased pseudo-*R*^2^ and *R*^2^ values to a greater degree than variable selection using point-biserial *r*. Moreover, ML may be useful for identifying unknown risk factors and unrelated factors. Our method may be generalized to other types of medical research because of the procedural simplicity of using top-ranked variables selected by SR via GP.

## Supplementary Information

Supplementary Information.

## Data Availability

The dataset analyzed and the programs developed in this study are available on GitHub as supplementary files (https://github.com/kiwindow/SymbolicRegression). The codes to perform SR via GP are written in Mathematica (Wolfram Research Inc., Champaign, IL), and DataModeler PRO or EDU (Evolved Analytics LLC, Rancho Santa Fe, CA) is needed. The program for SR via GP is uploaded as BuildModel.m along with the init.m file needed to import user-defined functions. The dataset is saved as newMaxGD2.xlsx and newMaxGD2.csv. Usage instructions are provided in README.md and ReadMeFirst.nb. The minimum commands explained in README.md are written on QuickStarte.nb file.
