# Supplementary material for: Machine learning for morbid glomerular hypertrophy
Source: Sci Rep. 2022 Nov 9;12:19155. doi: 10.1038/s41598-022-23882-7 (PMC9646707; doi:10.1038/s41598-022-23882-7)
Supplement: Supplementary file 1 — Supplementary Information. [file 41598_2022_23882_MOESM1_ESM.docx]

**Supplemental Material
Supplemental Material to “Predictive factor selection by machine learning for morbid glomerular hypertrophy”**

Yusuke Ushio ^1^, Hiroshi Kataoka ^1,2^*, Kazuhiro Iwadoh ^1,3^, Mamiko Ohara ^4^, Tomo Suzuki ^4^, Maiko Hirata ^5^, Shun Manabe ^1^, Keiko Kawachi ^1^, Taro Akihisa ^1^, Shiho Makabe ^1^, Masayo Sato ^1^, Naomi Iwasa ^1,2^, Rie Yoshida ^1,2^, Junichi Hoshino ^1^, Toshio Mochizuki ^1,2^, Ken Tsuchiya ^3^, and Kosaku Nitta ^1^

^1^ Department of Nephrology, Tokyo Women’s Medical University, Tokyo 162-8666, Japan

^2^ Clinical Research Division for Polycystic Kidney Disease, Department of Nephrology, Tokyo Women’s Medical University, Tokyo 162-8666, Japan

^3^ Department of Blood Purification, Tokyo Women’s Medical University, Tokyo 162-8666, Japan

^4^ Department of Nephrology, Kameda Medical Center, Chiba 296-8602, Japan

^5^ Japanese Red Cross Saitama Hospital, Saitama 330-8553, Japan

This appendix provides further methodological detail and results for the main paper.

**Supplemental Material**

**Supplemental Methods**

- Definition of comorbidities
- Pathological evaluation
- Permutation test

**Supplemental Table 1.** Top 20 variables in positive or negative correlation with the existence of MaxGD≥242.3 μm

**Supplemental Figures**

**Supplemental Fig. 1** The correlation coefficients (point-biserial *R*s) of 60 prognostic predictive variables with MaxGD≥242.3 μm. **Supplemental Fig. 2** The usage frequencies of 60 predictive variables in 1,819 predictive functions selected from 19,437 models that were generated by 43-fold cross-validation with leave-one-out using symbolic regression via genetic programming.

**Supplemental Methods**

**Definition of comorbidities**

The impacts of concomitant agent use and comorbidities at baseline were also assessed ^1^. The comorbidities were recorded as positive according to the criteria described next. Hypertension was defined as BP ≥140/90 mmHg and/or taking an antihypertensive agent. Hypercholesterolemia was defined as a serum TC level ≥220 mg/dL and/or taking an antidyslipidemic agent. Hypertriglyceridemia was defined as serum TG level ≥ 150 mg/dL and/or taking an antidyslipidemic agent. Hyperuricemia was defined as serum UA level ≥ 7.0 mg/dL and/or taking an antihyperuricemic agent.

**Pathological evaluation**

All kidney tissue specimens were obtained by percutaneous needle biopsy. Each specimen was evaluated for glomerular, interstitial, and vascular changes as previously described ^2,3^. The percentage of glomeruli that exhibited global sclerosis, segmental sclerosis, adhesions, and crescents were estimated. Mesangial cell proliferation, mesangial matrix expansion, interstitial fibrosis, interstitial inflammation, arteriosclerosis, and arteriolar hyalinosis in each patient were semi-quantitatively scored. The Oxford MEST-C criteria ^3-5^ was assessed by the following parameters: mesangial hypercellularity (M0: < 50% of glomeruli showing hypercellularity; M1: > 50% of glomeruli showing hypercellularity), endocapillary hypercellularity (E0: absent; E1: present), segmental glomerulosclerosis (S0: absent; S1: present), tubular atrophy/interstitial fibrosis (T0: < 25%; T1: 25–50%; T2: > 50% of cortical area involved), and cellular/fibrocellular crescents (C0: absent; C1: present in a least 1 but <25% of glomeruli; C2: present in at least 25% of glomeruli). We also assessed the maximal glomerular area (Max GA) and the MaxGD of the maximally-hypertrophied glomerulus (the largest renal corpuscle) identified in serial sections ^6^. MaxGD was calculated as the mean of two measurements, i.e., the maximal diameter of the maximal profile area in the largest renal corpuscle ^7^, and the maximal chord perpendicular to the maximal diameter in each specimen ^6^.

**Permutation test**

We performed a permutation test ^8,9^ using Python with libraries from scikit-learn *^10^* (<https://scikit-learn.org/stable/auto_examples/feature_selection/plot_permutation_test_for_classification.html#sphx-glr-auto-examples-feature-selection-plot-permutation-test-for-classification-py>) on the entire dataset.

**Supplementary References**

1. Ording, A. G. & Sorensen, H. T. Concepts of comorbidities, multiple morbidities, complications, and their clinical epidemiologic analogs. *Clinical epidemiology*. **5**, 199-203 (2013).

2. Kataoka, H., Ohara, M., Honda, K., Mochizuki, T. & Nitta, K. Maximal glomerular diameter as a 10-year prognostic indicator for IgA nephropathy. *Nephrology Dialysis Transplantation*. **26**, 3937-U3674 (2011).

3. Roberts, I. S. *et al.* The Oxford classification of IgA nephropathy: pathology definitions, correlations, and reproducibility. *Kidney international*. **76**, 546-556 (2009).

4. Trimarchi, H. *et al.* Oxford Classification of IgA nephropathy 2016: an update from the IgA Nephropathy Classification Working Group. *Kidney international*. **91**, 1014-1021 (2017).

5. Cattran, D. C. *et al.* The Oxford classification of IgA nephropathy: rationale, clinicopathological correlations, and classification. *Kidney international*. **76**, 534-545 (2009).

6. Kataoka, H., Ohara, M., Honda, K., Mochizuki, T. & Nitta, K. Maximal glomerular diameter as a 10-year prognostic indicator for IgA nephropathy. *Nephrology, dialysis, transplantation : official publication of the European Dialysis and Transplant Association - European Renal Association*. **26**, 3937-3943 (2011).

7. Lane, P. H., Steffes, M. W. & Mauer, S. M. Estimation of glomerular volume: a comparison of four methods. *Kidney international*. **41**, 1085-1089 (1992).

8. Valente, G., Castellanos, A. L., Hausfeld, L., De Martino, F. & Formisano, E. Cross-validation and permutations in MVPA: Validity of permutation strategies and power of cross-validation schemes. *Neuroimage*. **238**, 118145 (2021).

9. Ball, T. M., Squeglia, L. M., Tapert, S. F. & Paulus, M. P. Double Dipping in Machine Learning: Problems and Solutions. *Biol Psychiatry Cogn Neurosci Neuroimaging*. **5**, 261-263 (2020).

10. Abraham, A. *et al.* Machine learning for neuroimaging with scikit-learn. *Front Neuroinform*. **8**, 14 (2014).

11. Vabalas, A., Gowen, E., Poliakoff, E. & Casson, A. J. Machine learning algorithm validation with a limited sample size. *PloS one*. **14**, e0224365 (2019).

**Supplemental Table 1.** **Top 20 variables in positive or negative correlation with the existence of MaxGD≥242.3 μm**

|  | Variables | *R* |
| --- | --- | --- |
| 1 | BMI (kg/m^2^) | 0.530 |
| 2 | Arteriolosclerosis (grades 0–3) | 0.502 |
| 3 | Complement C3 (mg/dL) | 0.474 |
| 4 | U-Prot during 10-year follow-up (grades 0–4) | 0.460 |
| 5 | Body weight (kg) | 0.411 |
| 6 | Increase in dipstick hematuria during 10-year follow-up (vs. no) | 0.406 |
| 7 | Intimal thickening of the interlobular artery (grades 0–3) | 0.389 |
| 8 | Perihilar hyalinosis (vs. no) | 0.368 |
| 9 | Increase in U-Prot during 10-year follow-up (vs. no) | 0.345 |
| 10 | Hyperuricemia (vs. no) | 0.306 |
| 11 | Edema (vs. no) | −0.295 |
| 12 | Nephrotic syndrome (vs. no) | −0.295 |
| 13 | Total protein (g/dL) | 0.293 |
| 14 | Focal adhesions to Bowman's capsule (vs. no) | 0.270 |
| 15 | eGFR | −0.266 |
| 16 | C-reactive protein (mg/dL) | 0.263 |
| 17 | Hypertriglyceridemia (vs. no) | 0.244 |
| 18 | Serum albumin (g/dL) | 0.224 |
| 19 | Protein intake (g/kg・ideal body weight /day) | 0.224 |
| 20 | Fibrous crescent (%) | 0.218 |

Abbreviations: *R*, point-biserial correlation coefficient; BMI, body mass index; C3, component 3; U-Prot, Urinary protein excretion; eGFR, estimated glomerular filtration rate.


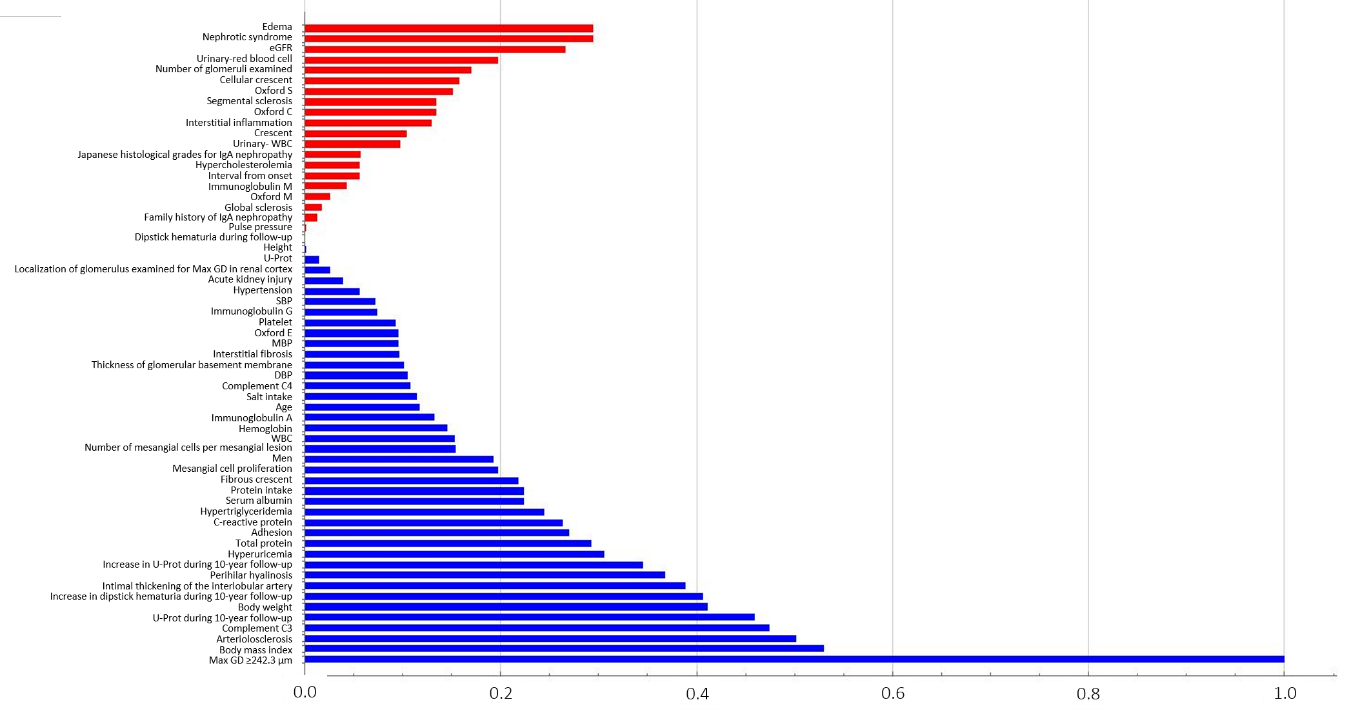


**Supplemental Fig. 1 The correlation coefficients (point-biserial *R*s) of 60 prognostic predictive variables with MaxGD≥242.3 μm.** Red column represents negative correlation and blue column represents positive correlation (listed in ascending order from top to bottom). Abbreviations: eGFR, estimated glomerular filtration rate; WBC, white blood cell; U-Prot, Urinary protein excretion; SBP, systolic blood pressure; MBP, mean blood pressure; DBP, diastolic blood pressure; Complement C4, complement component 4; Complement C3, complement component 3.


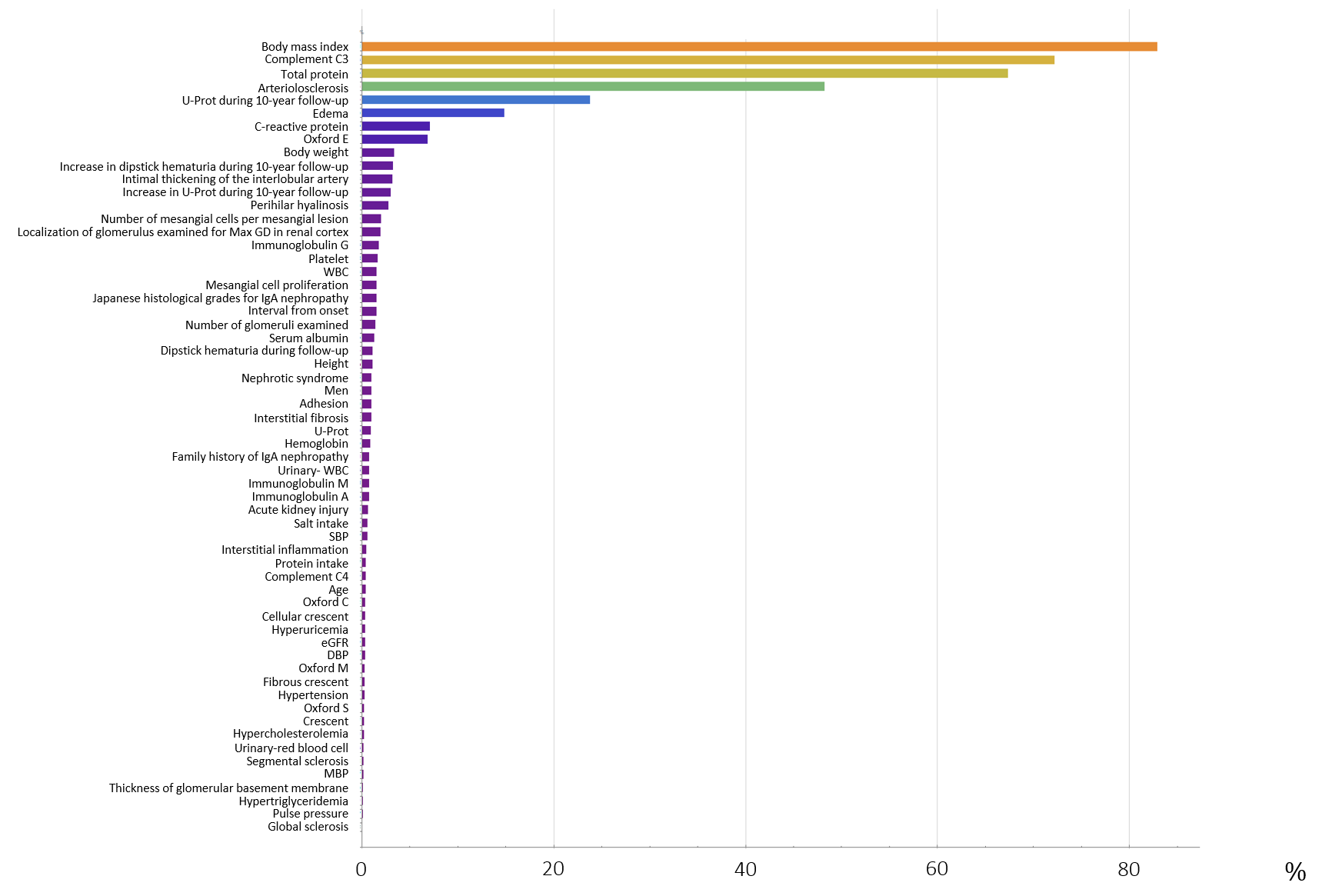


**Supplemental Fig. 2 The usage frequencies of 60 predictive variables in 1,819 predictive functions selected from 19,437 models that were generated by 43-fold cross-validation with leave-one-out using symbolic regression via genetic programming.** The frequencies of each predictive variable, which were used in the 1,819 predictive functions, are listed in descending order. The 60 variables were used at least once, and 5 variables were used in more than 20% of the predictive functions. Abbreviations: eGFR, estimated glomerular filtration rate; WBC, white blood cell; U-Prot, Urinary protein excretion; SBP, systolic blood pressure; MBP, mean blood pressure; DBP, diastolic blood pressure; Complement C4, complement component 4; Complement C3, complement component 3.
